# Development of an Online Health Care Assessment for Preventive Medicine: A Machine Learning Approach

**DOI:** 10.2196/18585

**Published:** 2020-06-05

**Authors:** Cheng-Sheng Yu, Yu-Jiun Lin, Chang-Hsien Lin, Shiyng-Yu Lin, Jenny L Wu, Shy-Shin Chang

**Affiliations:** 1 Department of Family Medicine Taipei Medical University Hospital Taipei Taiwan; 2 Department of Family Medicine School of Medicine College of Medicine, Taipei Medical University Taipei Taiwan

**Keywords:** machine learning, online healthcare assessment, medical informatics, preventive medicine

## Abstract

**Background:**

In the era of information explosion, the use of the internet to assist with clinical practice and diagnosis has become a cutting-edge area of research. The application of medical informatics allows patients to be aware of their clinical conditions, which may contribute toward the prevention of several chronic diseases and disorders.

**Objective:**

In this study, we applied machine learning techniques to construct a medical database system from electronic medical records (EMRs) of subjects who have undergone health examination. This system aims to provide online self-health evaluation to clinicians and patients worldwide, enabling personalized health and preventive health.

**Methods:**

We built a medical database system based on the literature, and data preprocessing and cleaning were performed for the database. We utilized both supervised and unsupervised machine learning technology to analyze the EMR data to establish prediction models. The models with EMR databases were then applied to the internet platform.

**Results:**

The validation data were used to validate the online diagnosis prediction system. The accuracy of the prediction model for metabolic syndrome reached 91%, and the area under the receiver operating characteristic (ROC) curve was 0.904 in this system. For chronic kidney disease, the prediction accuracy of the model reached 94.7%, and the area under the ROC curve (AUC) was 0.982. In addition, the system also provided disease diagnosis visualization via clustering, allowing users to check their outcome compared with those in the medical database, enabling increased awareness for a healthier lifestyle.

**Conclusions:**

Our web-based health care machine learning system allowed users to access online diagnosis predictions and provided a health examination report. Users could understand and review their health status accordingly. In the future, we aim to connect hospitals worldwide with our platform, so that health care practitioners can make diagnoses or provide patient education to remote patients. This platform can increase the value of preventive medicine and telemedicine.

## Introduction

In the ever-changing technological era, the internet can provide rapid and convenient medical services in the form of health care, preventive medicine, and telemedicine. Medical informatics is a multidisciplinary field that comprises medicine and computer science. As computer technology continues to advance, medical informatics can be used to develop various applications such as electronic medical records (EMRs), medical image processing, clinical diagnosis decision systems, hospital information management systems, telemedicine, and internet and health information systems [1-4].

To construct a health care information system, several factors must be considered: the hospital information system, including both clinical management and diagnosis services; the storage and processing of patient information, such as EMRs and electronic health records; decision support systems, such as expert diagnosis systems; and the artificial intelligence (AI) algorithms that need to be applied to those factors (eg, data mining in EMRs and decision-making in clinical diagnosis) [5-8].

The mass application of EMRs and the digitalization of medical equipment and instruments have led to the continuous expansion of information capacity in hospital databases. Therefore, informatics research should focus on basic electronic medical database construction, data collection and analysis, medical decision support, and automatic knowledge acquisition. Furthermore, the use of machine learning (ML) technology in AI to extract the most important information has led to cutting-edge research in medicine [9-13]. The goal of AI is to construct an intelligent machine that imitates the natural intelligence of humans. Computers, robots, and software that are made with such technology will have human-like thinking processes, but with the ability to utilize superhuman speed and power effectively. Knowledge engineering is an essential part of AI research, especially ML, because AI operations require a significant amount of real-world data.

ML is defined as a “machine that is capable of self-learning without any guidance.” Therefore, the main purpose of ML is to make computers self-learning and auto-correcting when analyzing data. The core technology of ML must identify specific patterns and information hidden within very large data sets using statistical analysis and prediction automatically [14-17].

Disease and disability are influenced by several factors: environmental factors, genetic predisposition, pathogens, and lifestyle choices. Some conditions are a dynamic process that can affect an individual before they are aware of any problem [18-20]. The core of preventive medicine is to prevent chronic diseases among people who are at risk of certain diseases. In some cases, it can also be used to reverse their condition, returning them to a good health status. In the past, due to information asymmetry, doctors and hospitals led the medical environment, and patients did not have access to any appropriate methods or information to implement real-time self-management. Patients who failed to obtain an early diagnosis would have to pay higher health care costs. Therefore, the spirit of prevention medicine is that “an ounce of prevention is worth a pound of cure” [21-23].

Metabolic syndrome (MetS) is a cluster of conditions comprising high blood sugar, high blood pressure, abnormal blood lipid levels, abdominal obesity, and other metabolic risk factors. It is a warning sign of potential future chronic disease. People with MetS have an increased risk of subsequent development of type II diabetes, hypertension, hyperlipidemia, heart disease, and stroke compared with healthy people [24-28].

Chronic kidney disease (CKD) is defined as kidney function that is impaired for longer than 3 months, leading to irreversible damage. The National Kidney Foundation Kidney Disease Outcome Quality Initiative guideline classifies CKD into 5 stages according to the estimated glomerular filtration rate (eGFR) and using the recommended Modification of Diet in Renal Disease (MDRD) equation [29]. There are many causes of CKD, such as congenital anomalies of the kidney, urinary tract obstruction, urinary tract infection, and glomerulopathy. In addition, hypertension, diabetes, and gout are common chronic diseases that cause CKD if undertreated [30,31].

Telemedicine uses information and telecommunication technology to deliver medical information and physicians’ diagnoses to patients without the limitations of time and space. It combines information and communication technologies with medical expertise to provide various services: remote consultation and conferencing for doctors; comprehensive medical care for residents in remote and outlying islands; and teaching and training opportunities for medical staff. The internet can be used to assist with the popularization of telemedicine to achieve a two-way communication channel between patients and medical practitioners [32,33]. Therefore, this study aims to construct an online ML-driven medical database system from EMRs of subjects who have undergone health examination, and provide online self-health evaluation for MetS and CKD.

## Methods

### Setting

The study was conducted at the Health Management Center (HMC) of Taipei Medical University Hospital (TMUH). Electronic medical records (EMRs) were obtained and reviewed from the HMC, which receives approximately 60 to 70 visits per month.

### Ethics

The study was approved by the Institutional Review Board (IRB) of TMUH prior to data collection (TMUH TMU-JIRB number N202003088), in accordance with the original and amended Declaration of Helsinki. The IRB waived the need for informed consent because of the retrospective nature of this study.

### EMR Database and System

**Figure 1:**
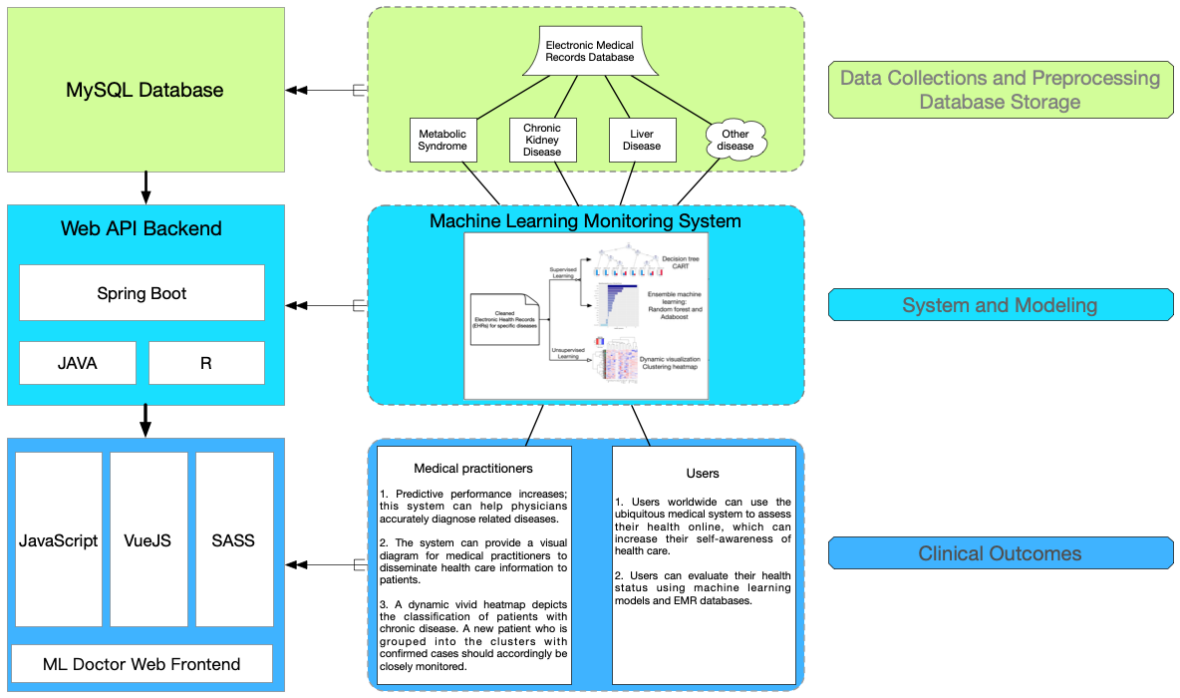
The structure of web-based machine learning medical system. API: application programming interface; EMR: electronic medical record; ML: machine learning.

The databases and the selected predicting variables (Table 1) were derived from previous publication on MetS and CKD [34-36]. Figure 1 shows an overview of the system and the main functions. Briefly, using a series of complicated procedures, the two databases (MetS and CKD) were connected to an internet platform to construct one integrated system. This web-based system was embedded with ML models to provide various medical evaluations and analyses. The online system was constructed on a server as a web-based environment. The frontend implementation included the programming language JavaScript (Oracle Corp), the framework VueJS (Vue), and the styling Syntactically Awesome Style Sheets (Sass). The backend implementation used Java and R as the programming languages, and all ML calculations and evaluations were conducted using the statistical program R (version 3.6.1, R Foundation for Statistical Computing). The back web framework was Spring Boot (Pivotal Software), connecting the MySQL (Oracle Corp) database as the storage system.

### Study Populations

**Figure 2:**
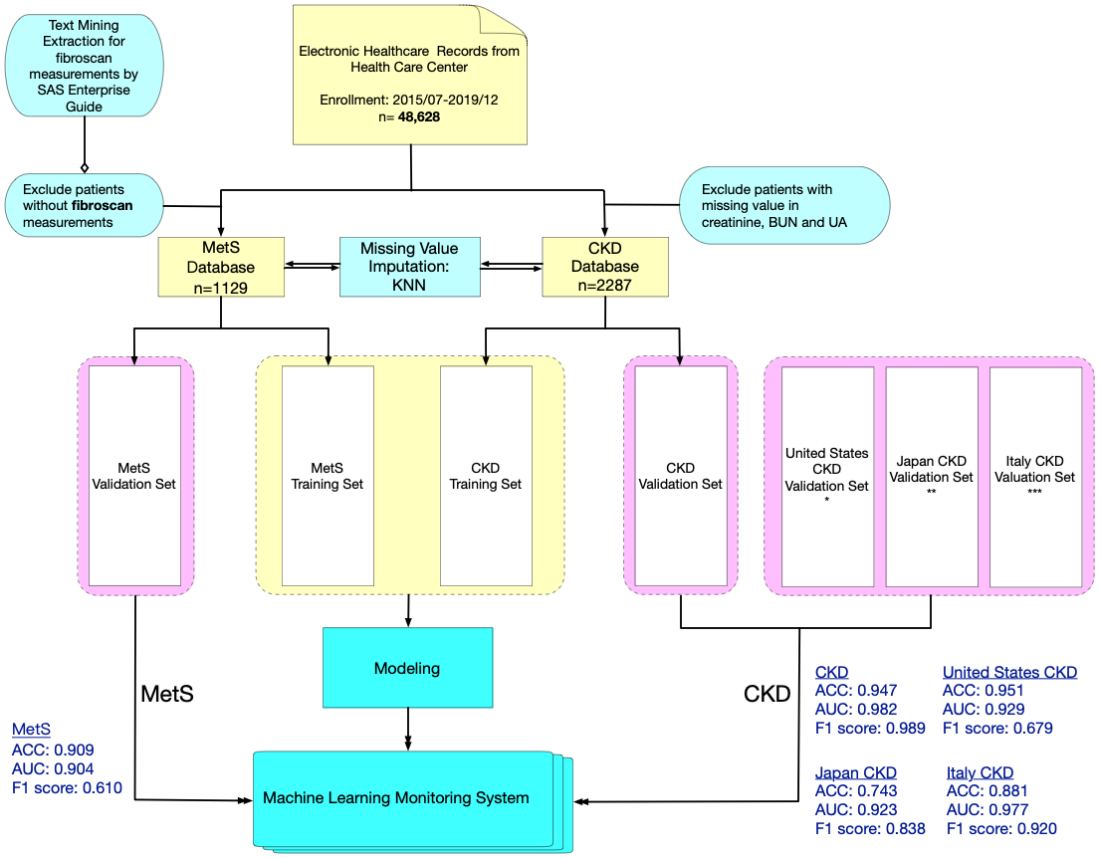
Flowchart of data collection and preprocessing for MetS and CKD data sets including training and validation sets. SAS Enterprise Guide is a software that combines the analytic ability of SAS software with a user-friendly interface. It provides several functions of Structured Query Language (SQL), which includes a text mining technique. ACC: accuracy; AUC: area under the curve; BUN: blood urea nitrogen; CKD: chronic kidney disease; KNN: k-nearest neighbors algorithm; MetS: metabolic syndrome; UA: uric acid. * Centers for Disease Control and Prevention (CDC) and National Center for Health Statistics (NCHS) [37], ** Iimori et al [39], *** De Nicola et al [38].

Figure 2 [37-39] shows an overview of the main study population and the validation populations. Briefly, the starting study population included 48,628 EMRs of Taiwanese adults aged over 18 years who underwent a self-paid health examination at TMUH from July 2015 to December 2019. All the study participants completed a self-questionnaire on demographics, existing medical conditions, and the use of medications.

**Table 1:** The list of predicting variables in the electronic health care records.

Disease and predicting variable		Unit
**Metabolic syndrome**		
	Sex	Male/Female
	Age	years
	Body mass index	kg/m^2^
	Waist circumference	cm
	Glutamic-oxaloacetic transaminase	IU/L
	Glutamate pyruvate transaminase	IU/L
	γ-Glutamyl transpeptidase	U/L
	Total bilirubin	mg/dL
	Alkaline phosphatase	IU/L
	Blood urea nitrogen	mg/dL
	Creatinine	mg/dL
	Uric acid	mg/dL
	Albumin	g/dL
	Cholesterol	mg/dL
	High-density lipoprotein	mg/dL
	Low-density lipoprotein	mg/dL
	Hemoglobin A_1c_	%
	Glucose AC	mg/dL
	Triglycerides	mg/dL
	Systolic blood pressure	mm Hg
	Diastolic blood pressure	mm Hg
	Elastic modulus (E) score	kPa
	Controlled attenuation parameter (CAP) score	dB/m
**Chronic kidney disease**		
	Sex	Male/Female
	Age	years
	Body mass index	kg/m^2^
	Waist circumference	cm
	Glutamic-oxaloacetic transaminase	IU/L
	Glutamate pyruvate transaminase	IU/L
	γ-Glutamyl transpeptidase	U/L
	Total bilirubin	mg/dL
	Alkaline phosphatase	IU/L
	Blood urea nitrogen	mg/dL
	Creatinine	mg/dL
	Uric acid	mg/dL
	Albumin	g/dL
	Cholesterol	mg/dL
	High-density lipoprotein	mg/dL
	Low-density lipoprotein	mg/dL
	Hemoglobin A_1c_	%
	Hypertension	Yes/No

Subsequently, the starting population data underwent data cleaning and preprocessing to form two distinct databases (MetS and CKD) for ML. For the MetS database, there were a total of 1129 participants after the exclusion of participants without FibroScan (Echosens) measurements. For the CKD database, there were a total of 2287 participants after the exclusion of participants without values for creatinine, blood urea nitrogen, and uric acid.

Due to the inconsistent definition of MetS across the world, the ML performance of the MetS database and the CKD database were validated using different study populations. The ML performance of the CKD database was validated using Taiwanese, Italian, US, and Japanese data sets, but the ML performance of the CKD database was only validated using a Taiwanese data set [37-39]. Since different variables may be unavailable in different validation data sets, unavailable variables were simply excluded in ML performance analysis for a balanced comparison.

### ML Techniques

The ML techniques used in this system included supervised learning models, such as classification and regression tree (CART) and random forest [35,36]. Supervised learning was applied to classify the patients in the training set and predict patients with a specific chronic disease or syndrome in the validation set before the prediction model was available on this system [40,41]. In addition, unsupervised learning (hierarchical clustering using the Ward method and Euclidean distance) was embedded in a heat map, providing classified visualization between new input records and the database. An interactive heat map that could be rearranged or zoomed in and out was applied to this system [42-47].

All outcomes were presented on the web platform after the ML system evaluated the users’ EMRs. Although the ML system was developed on a web-based interface, it could be embedded in the Internet of Medical Things (IoMT) environment, for example, as apps or real-time monitoring systems between several medical centers and hospitals [48-50].

### Questionnaire Selection

To measure the usability of websites, we invited potential users of the ML system (physicians, medical staff, and potential users) to fill out a system usability scale (SUS) evaluation questionnaire. SUS was chosen as the usability test tool because previous studies found it to be reliable and quick to answer, and the final score is provided with interpretation based on a well-established reference standard [51,52]. In general, the higher the SUS score, the better the usability of the website. Details about the questionnaire design (the 10 questions), score summary, and results of reliability and validity tests are given in Multimedia Appendix 1.

## Results

**Figure 3:**
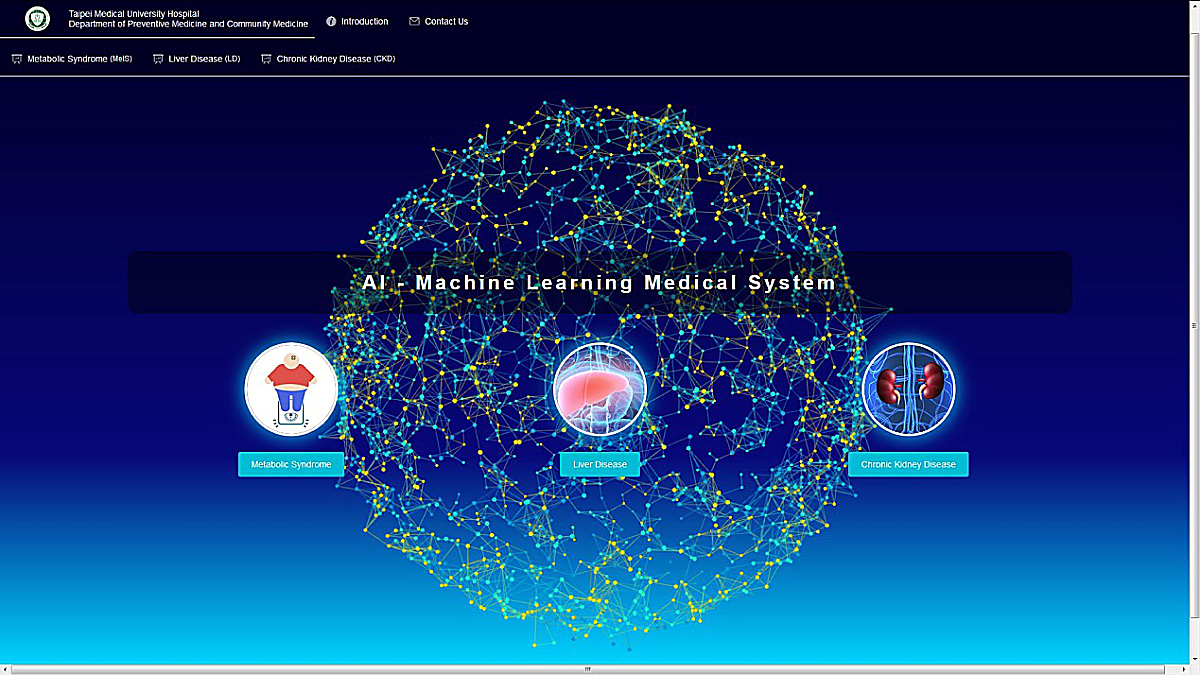
Home page of the machine learning health care system.

**Figure 4:**
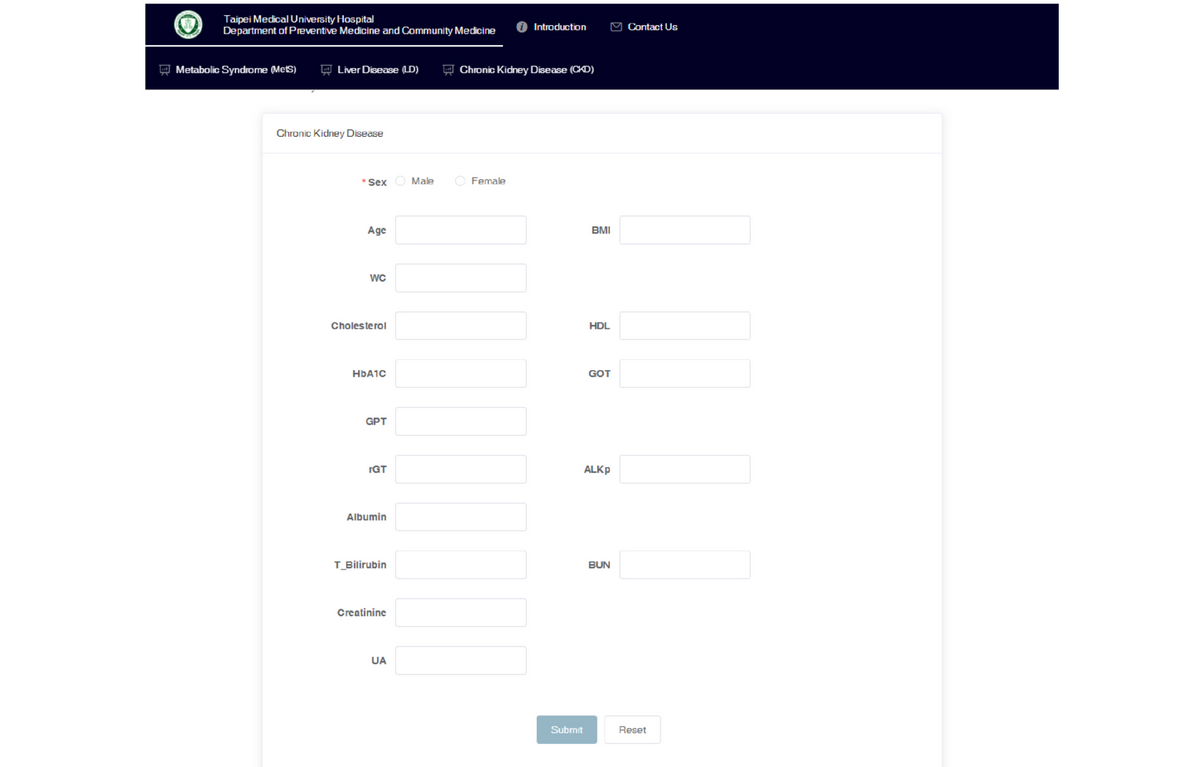
Interface of the input page for disease assessment.

The web-based health care ML system provides online diagnosis of three diseases (Figure 3), and it is available on the internet [53]. The website provides an assessment of MetS and CKD; the system for noncancer liver disease is still under beta testing. Report pages are provided for online diagnosis of each disease. Therefore, users from all over the world can choose the evaluation provided depending on their requirements. Users input the predicting variables (Table 1) into the website to evaluate their health (Figure 4), and the evaluation results will appear in <5 seconds when there is a single request. Missing predicting variables are allowed, and the missing values will be imputed based on the mean values from the database. However, the users are warned that missing predicting variables may result in poorer prediction accuracy. The details of stress tests with different numbers of requests (100 to 800) can be found in Multimedia Appendix 2. Briefly, a stress test with 800 requests reports a throughput of 4.7 requests per second. To evaluate the usability of the system, we invited 30 volunteers to complete the SUS evaluation questionnaire. The volunteers included 6 physicians, 12 medical staff, and 12 potential users (Multimedia Appendix 1). It was found that the average SUS score is 74, which indicates a good usability rating [54]. In addition, results were found to be reliable and valid by Kaiser-Meyer-Olkin and Bartlett tests. The entire analysis process follows a strict privacy policy, so that none of the patients’ private information is ever recorded.

**Figure 5:**
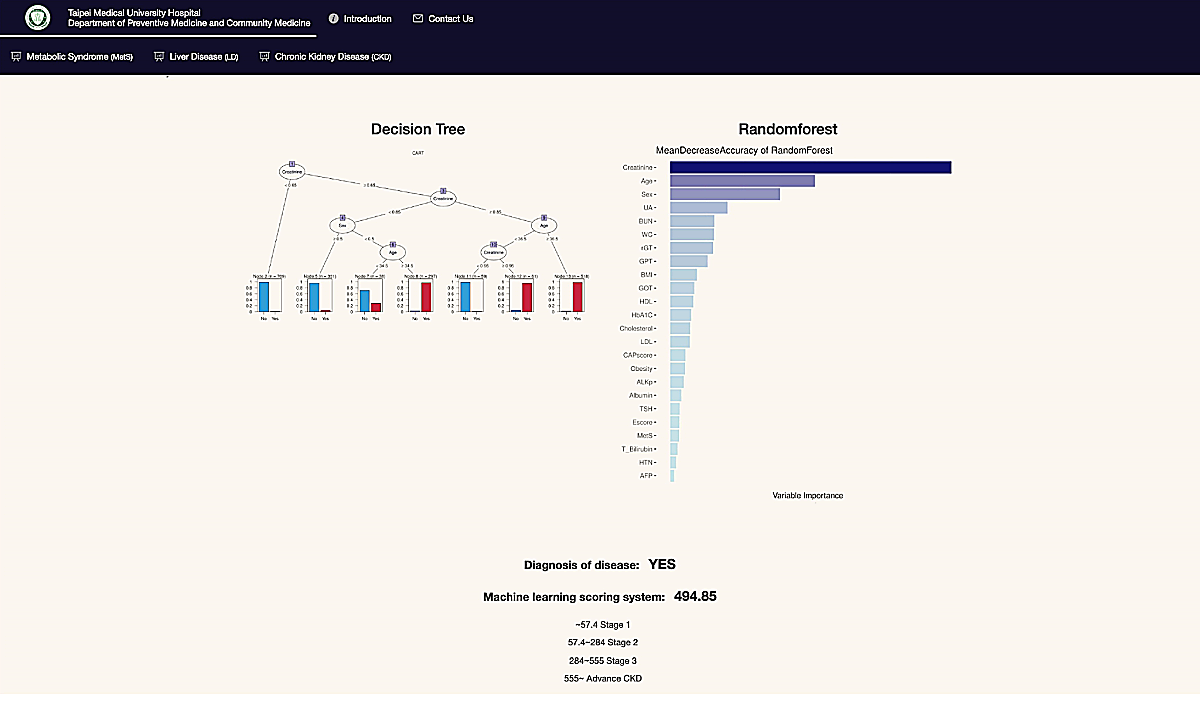
Outcome page for supervised learning models and the scoring system for disease diagnosis.

**Figure 6:**
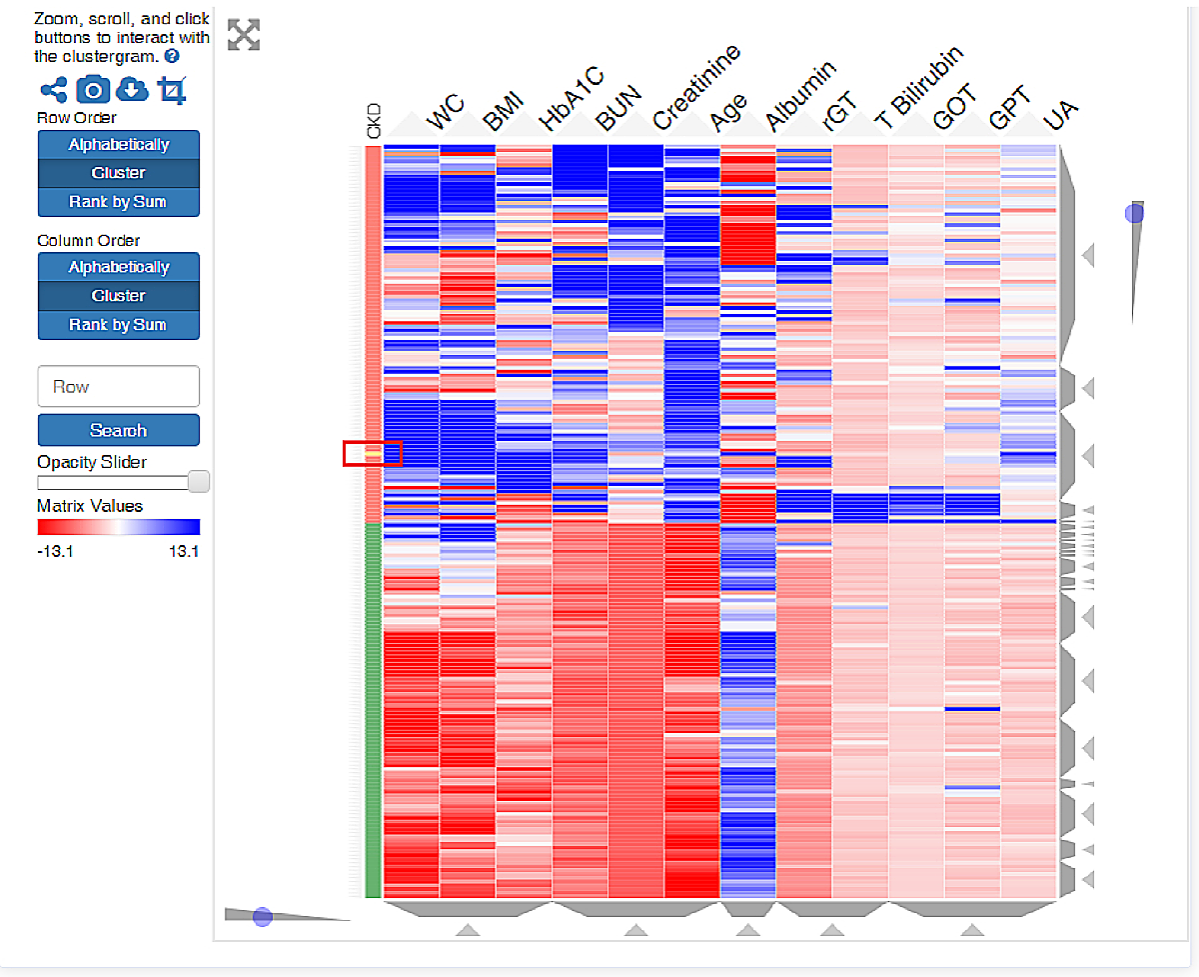
Dynamic interactive heat map obtained using unsupervised clustering. Green: healthy patients; orange: CKD patients; blue: normal values; red: abnormal values. The new patients (yellow bar in red rectangle) are compared and clustered into the system’s patient database.

The clinical outcomes established by our database are reported on the website when users have finished entering their medical record data on the website (Figure 5). The CART model and ensemble learning model (random forest) are shown in the output interface. A scoring prediction model obtained using the supervised learning model is also provided online. For unsupervised learning, a color visualization of the clustering heat map depicts a vivid medical pattern of the patient’s EMR data, and a record of each user is also constructed using hierarchical clustering with yellow highlights labeling in the heat map (Figure 6). The user will then be classified as more similar to either a healthy subject (green column on the lower left) or an unhealthy subject (orange column on the upper left). A blue bar depicts abnormal values, while a red bar depicts normal values. In addition, on the web system, users can choose to view it as landscape or portrait. The zoom-in and zoom-out functions and the height of the cluster are also dynamic, with users being able to change the settings online to inspect the medical outcomes in detail.

Characteristics of participants in the training and validation data set for MetS can be found in Table 2 and the characteristics of participants of the training set and the validation sets for CKD can be found in Table 3. In general, there are minimal differences in patient characteristics between the training data set and the validation data set for the Taiwanese population of MetS and CKD. However, when comparing the characteristics of the Taiwanese population with other populations (US, Italy, and Japan) for CKD ML performance validation, it was found that there are substantial differences in age and presence of hypertension (Table 3).

**Table 2:** Characteristics of participants in the training and validation data set for metabolic syndrome.

Characteristics		Training data set (n=904)		Validation data set (n=225)	
**Sex**					
	Female, n (%)		411 (45.5)		108 (48.0)
	Male, n (%)		493 (54.5)		117 (52.0)
Age, years, median (IQR)		44 (37-50.25)		43 (38-50)	
Body mass index, kg/m^2^, median (IQR)		23.6 (21.3-25.9)		22.9 (21.2-26)	
Waist circumference, cm, median (IQR)		81.75 (74.5-88)		80.5 (74-87)	
Albumin, g/dL, median (IQR)		4.6 (4.4-4.8)		4.6 (4.4-4.8)	
Alkaline phosphatase, IU/L, median (IQR)		58 (49-69)		58 (48-69)	
Glutamic-oxaloacetic transaminase, IU/L, median (IQR)		20 (17-24.25)		20 (17-25)	
Glutamate pyruvate transaminase, IU/L, median (IQR)		20 (14-31)		19 (14-28)	
Total bilirubin, mg/dL, median (IQR)		0.6 (0.5-0.9)		0.6 (0.4-0.8)	
γ-Glutamyl transpeptidase, U/L, median (IQR)		18 (12-27)		17 (11.55-25)	
Controlled attenuation parameter (CAP) score, dB/m, median (IQR)		247 (211-284)		241 (216-282)	
Elastic modulus (E) score, kPa, median (IQR)		4.2 (3.4-4.9)		4 (3.3-4.8)	
Blood urea nitrogen, mg/dL, median (IQR)		12 (10-15)		12 (10-14)	
Creatinine, mg/dL, median (IQR)		0.8 (0.6-0.9)		0.8 (0.6-0.9)	
Estimated glomerular filtration rate (eGFR) using the Modification of Diet in Renal Disease (MDRD) equation, median (IQR)		90.23 (80.49-104.77)		91.28 (82.72-107.26)	
Uric acid, mg/dL, median (IQR)		5.5 (4.5-6.6)		5.4 (4.3-6.6)	
Systolic blood pressure, mm Hg, median (IQR)		114 (105-125)		114 (105-126)	
Diastolic blood pressure, mm Hg, median (IQR)		73 (67-80)		72 (66-81)	
Cholesterol, mg/dL, median (IQR)		189 (165-209)		185 (168-209)	
Triglycerides, mg/dL, median (IQR)		90 (65-135.2)		86 (63-126)	
High-density lipoprotein, mg/dL, median (IQR)		55(45-67)		54 (47-66)	
Low-density lipoprotein, mg/dL, median (IQR)		123 (102-145)		123 (102-145)	
Hemoglobin A_1c_, %, median (IQR)		5.4 (5.2-5.6)		5.4 (5.2-5.5)	
Glucose AC, mg/dL, median (IQR)		91 (86-96)		90 (85-95)	

**Table 3:** Characteristics of participants in the training and validation data sets for chronic kidney disease.

Characteristics	Training data set (n=1830)	Validation data set, Taiwan (n=457)	Validation data set, United States (n=4434)	Validation data set, Italy (n=655)	Validation data set, Japan (n=996)
Sex, male, n (%)	902 (49.29)	209 (45.73)	2165 (48.83)	384 (58.63)	696 (69.88)
Chronic kidney disease, n (%)	164 (8.96)	38 (8.32)	410 (9.25)	523 (79.85)	919 (92.27)
Hypertension, n (%)	522 (28.52)	140 (30.63)	1730 (39.02)	599 (91.45)	908 (91.16)
Age, years, median (IQR)	46 (38-55)	45 (37-55)	53 (36-65)	67 (56-74.5)	70 (61-77)
Body mass index, kg/m^2^, median (IQR)	23.8 (21.4-26.4)	23.4 (21.3-26.2)	28.6 (24.8-33.5)	28.4 (25.8-31.6)	23.25 (21-25.8)
Waist circumference, cm, median (IQR)	82.5 (75.5-89.5)	81 (75-89)	99.5 (89-111.3)	—^a^	—
Glutamic-oxaloacetic transaminase, IU/L, median (IQR)	21 (17-26)	20 (17-25)	19 (16-24)	—	—
Glutamate pyruvate transaminase, IU/L, median (IQR)	20 (14-30)	19 (13-28)	18 (13-26)	—	—
γ-Glutamyl transpeptidase, U/L, median (IQR)	19 (13-30)	18 (12-33)	21 (15-33)	—	—
Total bilirubin, mg/dL, median (IQR)	0.6 (0.4-0.8)	0.6 (0.4-0.8)	0.4 (0.3-0.6)	—	—
Alkaline phosphatase, IU/L, median (IQR)	62 (51-76)	63 (50-78)	75 (62-91)	—	—
Blood urea nitrogen, mg/dL, median (IQR)	13 (11-16)	13 (10-15)	14 (11-18)	28 (21.2-37.3)	—
Creatinine, mg/dL, median (IQR)	0.8 (0.6-1.0)	0.7 (0.6-0.9)	0.85 (0.71-1.01)	1.49 (1.2-1.9)	1.8 (1.2-2.75)
Uric acid, mg/dL, median (IQR)	5.5 (4.5-6.7)	5.4 (4.5-6.5)	5.3 (4.4-6.4)	6.3 (5.2-7.6)	—
Albumin, g/dL, median (IQR)	4.6 (4.4-4.8)	4.6 (4.4-4.8)	4.1 (3.9-4.3)	4 (3.7-4.3)	4 (3.5-4.3)
Cholesterol, mg/dL, median (IQR)	186 (164-210)	185 (160-209)	185 (160-214)	189 (162.5-218)	—
High-density lipoprotein, mg/dL, median (IQR)	52 (44-64)	53 (43-64)	51 (42-61)	—	—
Low-density lipoprotein, mg/dL, median (IQR)	121 (100-145)	120 (100-142)	—	—	—
Hemoglobin A_1c_, %, median (IQR)	5.4 (5.2-5.6)	5.4 (5.2-5.7)	5.6 (5.3-6)	—	—

^a^Not available.

Table 4 shows the validation performances of supervised learning models in predicting MetS and CKD. In general, it was found that the random forest ML model has higher accuracy than the CART model. Using the random forest ML model, MetS can be predicted with an accuracy of 0.909, and CKD can be predicted up to an accuracy of 0.947. Due to the inconsistent definition of MetS globally, the ML performance of the MetS database has only been validated using the Taiwan data set. However, the ML performances of the CKD database have been validated using data sets from Taiwan, Italy, the United States, and Japan. In general, the CKD database shows good external applicability, and has high AUC for all 4 validation data sets (Taiwan: AUC=0.982; USA: AUC=0.929; Italy: AUC=0.977; Japan: AUC=0.923). However, the validation accuracy and F1 value of CKD prediction differs more substantially, as the unavailable data were excluded from the analysis. When compared to the Taiwanese CKD data set, the respective unavailable data are approximately 6% for the US data set, 50% for the Italy data set, and 67% for the Japan data set. Therefore, it is observed that the Japanese validation data set has the lowest accuracy (0.743) in predicting CKD, as it also has the highest proportion of unavailable data.

**Table 4:** The performance of supervised learning models on predicting metabolic syndrome and chronic kidney disease.

Model and disease		Accuracy	Area under the curve (AUC)	F1 score
**Classification and regression tree (CART)**				
	Metabolic syndrome (Taiwan)	0.874	0.887	0.448
	Chronic kidney disease (Taiwan)	0.945	0.928	0.965
**Random forest**				
	Metabolic syndrome (Taiwan)	0.909	0.904	0.610
	Chronic kidney disease (Taiwan)	0.947	0.982	0.989
	Chronic kidney disease (United States)	0.951	0.929	0.679
	Chronic kidney disease (Italy)	0.881	0.977	0.920
	Chronic kidney disease (Japan)	0.743	0.923	0.838

## Discussion

### Overview

This ML medical system for three common diseases in family medicine (MetS, CKD, and liver diseases) was constructed from EMR subjects who underwent self-paid health examination. Several ML prediction models are applied to the databases, and the outcomes are summarized and presented visually on the website for users and medical staff. The accuracy of predicting MetS reached 90.9%, and AUC was 0.904 in this system. For chronic kidney disease, the prediction accuracy reached 94.7%, and the AUC was 0.982. In general, users who were invited to test this system rated it with good usability and could easily assess their health online through this web-based ML monitoring system.

### CART

Decision trees are an important type of ML algorithm for predictive modeling. They are commonly used in data mining with the objective of creating a model that predicts the dependent variable (the target) based on numerous independent variables [34,37].

A decision tree is a nonparametric ML modeling technique used for regression and classification problems. In classification problems, the target variable is categorical, and the tree is used to identify which group or class a target variable would likely fall into. In regression problems, the target variable is continuous, and the tree is used to predict its value. To find solutions, a decision tree makes a sequential, hierarchical decision about the outcome’s variable according to the predictor [55-57].

Hence, CART can provide a visual tree-based diagram for medical practitioners to disseminate health care information to patients. It also helps users to understand the significance of different risk factors for specific diseases. For example, the cut-off controlled attenuation parameter (CAP) score was used to separate patients with MetS and those with other health observations. The CAP score was brought to the attention of users, thereby increasing their awareness of self-health [34].

### Random Forest

Random forest, also called random decision forests, is a popular ensemble learning method in ML. Ensemble methods use multiple learning algorithms to improve ML results by combining several decision tree models. This approach allows better predictive performance compared with a single model. Random forest is a parallel ensemble method in which the base learners are generated in parallel. The basic motivation of parallel methods is to exploit independence between the base learners because the error can be reduced dramatically by averaging [58,59]. As random forest provides a bagging technique for feature estimates, it also offers efficient estimates of the test error without incurring the cost of repeated model training associated with cross-validation. Moreover, random forest ranks risk factors in prediction models, which clinicians can use as a reference for diagnosis, and remote users can use to review their risk assessment of related diseases [34,60-62]. For instance, clinicians can refer to significant factors of certain diseases to determine whether those factors exceed the thresholds or not, allowing patients to be more vigilant about their risk of developing such diseases. In addition, sequential ensemble methods such as AdaBoost and XGBoost will be implemented and uploaded to our system in the future.

### Clustering

Hierarchical clustering is a widely used unsupervised learning technique that groups data with similar characteristics. Both agglomerative and divisive approaches use dendrograms for the results. A heat map is a color graphical representation of data, which uses a matrix with color gradients to present the similarity of data.

Many studies on genetic bioinformatics and bacterial ecology have used heat maps for the analysis of large and complicated data sets, and some medical studies have used heat maps with clustering to present the relationship between various biomarkers according to their characteristics [34,63-65]. Furthermore, our system provides an interactive clustering heat map for health care. From the perspective of big data, users can evaluate their health status by using ML models and EMR databases. In addition to online health evaluation, in the future, this system could be implemented into different IoMT to assist medical practitioners in achieving real-time health evaluations and monitoring remote patients or patients in specific wards. For the heat map, the EMR data of users were grouped into clusters of patients with diseases in the database; they would then be classified as clinically high-risk objects requiring close attention in the clinical setting [34]. Therefore, whether it is applied in preventive medicine for health management, in a monitoring system for critical care, or in the telemedicine environment, our system can provide real-time monitoring and help predict patient conditions.

### Limitations and Future Work

To the best of our knowledge, this is the first web-based machine learning system based on self-paid health examination subjects that can provide an online self-health evaluation for several common diseases (MetS, CKD, and liver diseases). The version 1.0 web-based system still has several limitations that may be improved in the next update. First, the 1.0 system is not yet ready for embedding into a hospital for real-time assessment. We are currently working on an improved system to accept unstructured data input and multimodal data, which are especially essential for the prediction of eye diseases such as macular degeneration. Second, the 1.0 system did not have a user login or account security function. Retrievable prediction and security will be improved as the system is matured for hospital embedment. Third, the 1.0 system does not have whole dynamic analyses such as an interactive decision tree; whole dynamic analyses will be incorporated in subsequent versions to improve communication between the medical staff and patients.

In the future, more clustering algorithms will be implemented in subsequent versions to make the prediction results more robust and reliable. Although the 1.0 system can currently only evaluate three chronic diseases (MetS, CKD, and liver diseases) frequently encountered in family medicine, more chronic disease prediction models, such as those for coronary artery disease, will be added in the near future.

### Conclusion

We constructed an ML health monitoring system to offer an online health assessment service to medical units, telemedicine patients, and all health-conscious users worldwide. Our aim is that this system will be implemented in medical centers as a real-time patient monitoring system and provide regular health evaluations for telemedicine patients. Online users can now access our platform and use ML technology to estimate their health status, increasing self-health awareness.

## Appendix

Multimedia Appendix 1 Questionnaire information.

Multimedia Appendix 2 Heat map clustering example and system loading test results.

## Abbreviations

AI: artificial intelligence
AUC: area under the curve
CAP: controlled attenuation parameter
CART: classification and regression tree
CKD: chronic kidney disease
eGFR: estimated glomerular filtration rate
EMRs: electronic medical records
HMC: Health Management Center
IoMT: Internet of Medical Things
IRB: institutional review board
MDRD: Modification of Diet in Renal Disease
MetS: metabolic syndrome
ML: machine learning
ROC: receiver operating characteristic
TMUH: Taipei Medical University Hospital
