# Correction: Development of an Online Health Care Assessment for Preventive Medicine: A Machine Learning Approach

**DOI:** 10.2196/21753

**Published:** 2020-07-27

**Authors:** Cheng-Sheng Yu, Yu-Jiun Lin, Chang-Hsien Lin, Shiyng-Yu Lin, Jenny L Wu, Shy-Shin Chang

**Affiliations:** 1 Department of Family Medicine Taipei Medical University Hospital Taipei Taiwan; 2 Department of Family Medicine School of Medicine College of Medicine, Taipei Medical University Taipei Taiwan

In “Development of an Online Health Care Assessment for Preventive Medicine: A Machine Learning Approach” (J Med Internet Res 2020;22(6):e18585) the authors noticed an error in the Institutional Review Board (IRB) approval number.

Under the Ethics heading in the Methods section, the IRB approval number was listed as “N201906023”

(TMUH TMU-JIRB number N201906023)

The correct number is “N202003088”:

(TMUH TMU-JIRB number N202003088)

The correction will appear in the online version of the paper on the JMIR Publications website on July 27, 2020, together with the publication of this correction notice. Because this was made after submission to PubMed, PubMed Central, and other full-text repositories, the corrected article has also been resubmitted to those repositories.

