# Supplementary material for: Development of an Online Health Care Assessment for Preventive Medicine: A Machine Learning Approach
Source: J Med Internet Res. 2020 Jun 5;22(6):e18585. doi: 10.2196/18585 (PMC7305560; doi:10.2196/18585)
Supplement: Multimedia Appendix 1 [file jmir_v22i6e18585_app1.pdf]

There are 30 feedbacks of questionnaires for our web-based machine learning health care system, including physicians, medical staffs and other people.

The System Usability Scale (SUS) is undoubtedly the most frequently used questionnaire to measure usability. It was created by John Brooke in 1986. In UX, SUS is always used in an online survey or after the each usability testing session for users to fill in. While SUS is frequently used today to measure the usability of websites, its usage is not limited to websites. It can be used to measure any systems and applications ranging from digital products such as mobile apps, digital kiosks, laptops to machinery. Apart from SUS, there are also other templates of questionnaire available to measure usability. The main reasons SUS is still preferred are:

1. SUS is quick. The template is readily available. There's no need to rephrase or think of your own questionnaire.
2. SUS is cheap. This questionnaire can be administered easily without much effort.
3. SUS is tested and reliable.

Although this 10-item scale looks simple, it has been validated with thousands of surveys over hundreds of studies for decades. Its results are intuitive and solid in convincing management to get things done. The System Usability Scale (SUS) a reliable, low-cost usability scale that can be used for global assessments of systems usability. The average SUS score is 68, which means the adjective rating is okay at 50th percentile. The score between 68 and 80.3 is Good with grade B, and SUS score > 80.3 is excellent.

The section 4 in this questionnaire is designed by SUS. The following is the summary of SUS score and the test of reliability and validity:

The statistical description of our 30 Questionnaires for SUS score

Statistics	SUS score
Minimum	57.5
Q1	67.5
Median	72.5
Mean	74.25
Q3	80
Maximum	97.5

Kaiser-Meyer-Olkin (KMO) Test is a measure of how suited your data is for Factor Analysis. The test measures sampling adequacy for each variable in the model and for the complete model. The statistic is a measure of the proportion of variance among variables that might be common variance. The lower the proportion, the more suited your data is to Factor Analysis. KMO values less than 0.6 indicate the sampling is not adequate and that remedial action should be taken. Some authors put this value at 0.5, so use your own judgment for values between 0.5 and 0.6.

Bartlett's test for Sphericity compares your correlation matrix (a matrix of Pearson correlations) to the identity matrix. In other words, it checks if there is a redundancy between variables that can be summarized with some factors.

Factor Analysis for Questionnaire for Section 4

KMO and Bartlett's Test		
Kaiser-Meyer-Olkin Measure of Sampling Adequacy		**0.625** (>0.6)
Bartlett's Test of Sphericity	Approximate Chi-Square	88.256
	degree of freedom	45
	significance	**<0.001**

Item	Factor 1	Factor 2	Factor 3
Q4-1		0.829	
Q4-2	0.877		
Q4-3		0.668	
Q4-4	0.723		
Q4-5		0.561	
Q4-6	0.776		
Q4-7		0.693	
Q4-8	0.527		0.607
Q4-9			-0.834
Q4-10			0.648

Reliability test
For factor 1 (Q4-2, Q4-4, Q4-6, Q4-8)
Cronbach's alpha = 0.743

For factor 2 (Q4-1, Q4-3, Q4-5, Q4-7)
Cronbach's alpha = 0.681

Factor Analysis for Questionnaire for Section 5

KMO and Bartlett's Test		
Kaiser-Meyer-Olkin Measure of Sampling Adequacy		**0.601** >(0.6)
Bartlett's Test of Sphericity	Approximate Chi-Square	104.328
	degree of freedom	21
	significance	**<0.001**

Item	Factor 1	Factor 2
Q5-1		0.936
Q5-2	0.924	
Q5-3		0.957
Q5-4	0.853	
Q5-5	0.866	
Q5-6	0.855	
Q5-7		0.949

Reliability test
Cronbach's alpha = 0.857

For factor 1 (Q5-2, Q5-4, Q5-5, Q5-6)
Cronbach's alpha = 0.906

For factor 2 (Q5-1, Q5-3, Q5-7)
Cronbach's alpha = 0.954

In factor analysis, the structure of factor in Q4 is followed by the design of SUS questionnaire. And in Q5, the Q5-2, Q5-4,Q5-5 and Q5-6 are in the same factor loading, which is about the communication between medical staffs and patients. Considering the remaining questions in Q5 (Q5-1, Q5-3 and Q5-7), the factor loading is about the contribution of IoMT in practice.

Furthermore, most physician respondents are positive and optimistic for the implementation of this machine learning healthcare assessment system in clinic. But some suggested that it is better to use only as a communication bridge when medical staff give healthcare education to patients.

We randomly select some questionnaire samples as follows:

ML-Doctor 網站使用滿意度

No: 5

1. 請問您是第一次造訪我們網站嗎？

☒是　　☐否

2. 您的身分是

☒醫師(請完成第 4、5、6 項)

☐臨床醫護人員(請完成第 4、5 項)

☐非臨床醫護人員

3. 年齡:

☐ 18-24

☐ 25-34

☐ 35-44

☐ 45-54

☒ 55-64

☐ 65 以上

4. MLdoctor 網站體驗與觀感調查，請依據問卷題目在方框內打勾

問卷題目	非常不同意	不同意	普通	同意	非常同意
1. 我想我會願意經常使用這個網站。				✓	
2. 我覺得這個網站過於複雜。			✓		
3. 我認為這個網站很容易使用。				✓	
4. 我想我需要有人幫助才能使用這個網站。		✓			
5. 我覺得這個網站的功能整合得很好。				✓	
6. 我覺得這個網站有太多不一致的地方。		✓			
7. 我可以想像大部份的人很快就可以學會使用這個網站。				✓	
8. 我覺得這個網站使用起來很麻煩。		✓			
9. 我很有自信能使用這個網站。				✓	
10. 我需要學會很多額外的資訊，才能使用這個網站。		✓			

5. 醫護人員 MLdoctor 網站體驗調查(如第一項勾選醫師及醫護人員者請繼續作答)

問卷題目	非常不同意	不同意	普通	同意	非常同意
1. 這個網站提供一個合適的管道來傳遞健康照護資訊					✓
2. 這個網站在我執行健康照護上會有幫助					✓
3. 這個網站改善了我提供醫療保健服務的途徑				✓	
4. 這個網站讓我更有效率地管理病患的健康				✓	
5. 這個網站讓我跟我的病患溝通更方便					✓
6. 這個網站讓我有更多機會跟我的病患互動					✓
7. 這個網站提供一個合適的管道來提供醫療保健服務如: 獲得教育資料，追蹤自己的活動並進行自我評估。				✓	

6. 臨床醫師 MLdoctor 網站體驗調查(如第一項勾選醫師請繼續作答)

I. 您每個月的看診人數大約為

- ☐ 未看診
- ☐ <20 人
- ☐ 20-100 人
- ☐ 100-200 人
- ☒ >200 人

II. 請預估上個月用網路搜尋健康資訊的病患大約比例為

- ☐ <1%
- ☐ 1-2%
- ☐ 3-5%
- ☐ 6-10%
- ☒ >10%
- ☐ 不確定

III. 您認為網路上的健康資訊的總體品質為

- ☐ 通常可靠
- ☐ 有时可靠
- ☒ 有时不可靠
- ☐ 通常不可靠
- ☐ 不知道

IV. **您有任何病患因查閱網路上的資訊而獲得健康上的好處嗎**

- [x] 有
- [ ] 沒有
- [ ] 不確定

V. **您的患者獲得了哪些好處？ [可複選]**

- [ ] 訂購有用的藥物或其他保健產品
- [ ] 從（聲稱的）實踐者那裡獲得有用的第二意見
- [x] 獲得有用的風險估計
- [ ] 從病友資訊協助網站獲得有用的建議
- [x] 儘早尋求適當的醫療幫助
- [x] 更好地了解他們的狀況
- [ ] 獲得社會對他們狀況的支持
- [ ] 其他:____.

VI. **總體來說，使用網路對您和患者的健康服務有什麼好處？ [可複選]**

- [x] 患者更有能力應對症狀或疾病
- [x] 諮詢時間更短
- [x] 患者對自我保健更有信心
- [ ] 患者在不需要時不尋求醫療幫助
- [x] 患者會盡快接受必要的檢查或治療
- [ ] 減少不必要的檢查
- [ ] 減少不必要的治療
- [ ] 其他:______________________

VII. **總體而言，您會如何描述患者對網路健康資料的體驗？**

- [x] 很好
- [ ] 好
- [ ] 不確定
- [ ] 不好
- [ ] 極差

VIII. **您是否會推薦病患使用本機器學習健康評估網站 MLdoctor**

- [x] 願意
- [ ] 不願意，原因:___________________
- [ ] 沒意見，對醫療資訊與互聯網不熟悉

您的 Email (選填): ____________________________________，如有任何相關意見諮詢，可透過 e-mail 聯繫您

謝謝您的寶貴意見，祝您身體健康，事業順利，闔府平安！

ML-Doctor 網站使用滿意度

No: 7

1. 請問您是第一次造訪我們網站嗎？

☑是　　　☐否

2. 您的身分是

☐醫師(請完成第 4、5、6 項)
☐臨床醫護人員(請完成第 4、5 項)
☑非臨床醫護人員

3. 年齡:

☐ 18-24
☐ 25-34
☑ 35-44
☐ 45-54
☐ 55-64
☐ 65 以上

4. MLdoctor 網站體驗與觀感調查，請依據問卷題目在方框內打勾

問卷題目	非常不同意	不同意	普通	同意	非常同意
1. 我想我會願意經常使用這個網站。					✓
2. 我覺得這個網站過於複雜。	✓				
3. 我認為這個網站很容易使用。				✓	
4. 我想我需要有人幫助才能使用這個網站。	✓				
5. 我覺得這個網站的功能整合得很好。					✓
6. 我覺得這個網站有太多不一致的地方。	✓				
7. 我可以想像大部份的人很快就可以學會使用這個網站。				✓	
8. 我覺得這個網站使用起來很麻煩。			✓		
9. 我很有自信能使用這個網站。				✓	
10. 我需要學會很多額外的資訊，才能使用這個網站。					✓

5. 醫護人員 MLdoctor 網站體驗調查(如第一項勾選醫師及醫護人員者請繼續作答)

問卷題目	非常不同意	不同意	普通	同意	非常同意
1. 這個網站提供一個合適的管道來傳遞健康照護資訊					
2. 這個網站在我執行健康照護上會有幫助					
3. 這個網站改善了我提供醫療保健服務的途徑					
4. 這個網站讓我更有效率地管理病患的健康					
5. 這個網站讓我跟我的病患溝通更方便					
6. 這個網站讓我有更多機會跟我的病患互動					
7. 這個網站提供一個合適的管道來提供醫療保健服務如: 獲得教育資料，追蹤自己的活動並進行自我評估。					

6. 臨床醫師 MLdoctor 網站體驗調查(如第一項勾選醫師請繼續作答)

I. 您每個月的看診人數大約為

☐ 未看診
☐ <20 人
☐ 20-100 人
☐ 100-200 人
☐ >200 人

II. 請預估上個月用網路搜尋健康資訊的病患大約比例為

☐ <1%
☐ 1-2%
☐ 3-5%
☐ 6-10%
☐ >10%
☐ 不確定

III. 您認為網路上的健康資訊的總體品質為

☐ 通常可靠
☐ 有时可靠
☐ 有时不可靠
☐ 通常不可靠
☐ 不知道

IV. 您有任何病患因查閱網路上的資訊而獲得健康上的好處嗎

- ☐ 有
- ☐ 沒有
- ☐ 不確定

V. 您的患者獲得了哪些好處？ [可複選]

- ☐ 訂購有用的藥物或其他保健產品
- ☐ 從（聲稱的）實踐者那裡獲得有用的第二意見
- ☐ 獲得有用的風險估計
- ☐ 從病友資訊協助網站獲得有用的建議
- ☐ 儘早尋求適當的醫療幫助
- ☐ 更好地了解他們的狀況
- ☐ 獲得社會對他們狀況的支持
- ☐ 其他:______________________________________.

VI. 總體來說，使用網路對您和患者的健康服務有什麼好處？ [可複選]

- ☐ 患者更有能力應對症狀或疾病
- ☐ 諮詢時間更短
- ☐ 患者對自我保健更有信心
- ☐ 患者在不需要時不尋求醫療幫助
- ☐ 患者會盡快接受必要的檢查或治療
- ☐ 減少不必要的檢查
- ☐ 減少不必要的治療
- ☐ 其他:__________________

VII. 總體而言，您會如何描述患者對網路健康資料的體驗？

- ☐ 很好
- ☐ 好
- ☐ 不確定
- ☐ 不好
- ☐ 極差

VIII. 您是否會推薦病患使用本機器學習健康評估網站 MLdoctor

- ☐ 願意
- ☐ 不願意，原因:_______________
- ☐ 沒意見，對醫療資訊與互聯網不熟悉

您的 Email (選填): ____________________________，如有任何相關意見諮詢，可透過 e-mail 聯繫您

謝謝您的寶貴意見，祝您身體健康，事業順利，闔府平安！

ML-Doctor 網站使用滿意度

No: 12

1. 請問您是第一次造訪我們網站嗎？

☐是　　☑否

2. 您的身分是

☐醫師(請完成第 4、5、6 項)
☑臨床醫護人員(請完成第 4、5 項)
☐非臨床醫護人員

3. 年齡:

☐ 18-24
☐ 25-34
☐ 35-44
☑ 45-54
☐ 55-64
☐ 65 以上

4. MLdoctor 網站體驗與觀感調查，請依據問卷題目在方框內打勾

問卷題目	非常不同意	不同意	普通	同意	非常同意
1. 我想我會願意經常使用這個網站。					✓
2. 我覺得這個網站過於複雜。		✓			
3. 我認為這個網站很容易使用。				✓	
4. 我想我需要有人幫助才能使用這個網站。		✓			
5. 我覺得這個網站的功能整合得很好。					✓
6. 我覺得這個網站有太多不一致的地方。		✓			
7. 我可以想像大部份的人很快就可以學會使用這個網站。				✓	
8. 我覺得這個網站使用起來很麻煩。	✓				
9. 我很有自信能使用這個網站。					✓
10. 我需要學會很多額外的資訊，才能使用這個網站。	✓				

5. 醫護人員 MLdoctor 網站體驗調查(如第一項勾選醫師及醫護人員者請繼續作答)

問卷題目	非常不同意	不同意	普通	同意	非常同意
1. 這個網站提供一個合適的管道來傳遞健康照護資訊				✓	
2. 這個網站在我執行健康照護上會有幫助				✓	
3. 這個網站改善了我提供醫療保健服務的途徑				✓	
4. 這個網站讓我更有效率地管理病患的健康		✓			
5. 這個網站讓我跟我的病患溝通更方便		✓			
6. 這個網站讓我有更多機會跟我的病患互動				✓	
7. 這個網站提供一個合適的管道來提供醫療保健服務如: 獲得教育資料，追蹤自己的活動並進行自我評估。					✓

6. 臨床醫師 MLdoctor 網站體驗調查(如第一項勾選醫師請繼續作答)

I. 您每個月的看診人數大約為
- ☐ 未看診
- ☐ <20 人
- ☐ 20-100 人
- ☐ 100-200 人
- ☐ >200 人

II. 請預估上個月用網路搜尋健康資訊的病患大約比例為
- ☐ <1%
- ☐ 1-2%
- ☐ 3-5%
- ☐ 6-10%
- ☐ >10%
- ☐ 不確定

III. 您認為網路上的健康資訊的總體品質為
- ☐ 通常可靠
- ☐ 有时可靠
- ☐ 有时不可靠
- ☐ 通常不可靠
- ☐ 不知道

IV. 您有任何病患因查閱網路上的資訊而獲得健康上的好處嗎

- ☐ 有
- ☐ 沒有
- ☐ 不確定

V. 您的患者獲得了哪些好處？[可複選]

- ☐ 訂購有用的藥物或其他保健產品
- ☐ 從（聲稱的）實踐者那裡獲得有用的第二意見
- ☐ 獲得有用的風險估計
- ☐ 從病友資訊協助網站獲得有用的建議
- ☐ 儘早尋求適當的醫療幫助
- ☐ 更好地了解他們的狀況
- ☐ 獲得社會對他們狀況的支持
- ☐ 其他:____.

VI. 總體來說，使用網路對您和患者的健康服務有什麼好處？[可複選]

- ☐ 患者更有能力應對症狀或疾病
- ☐ 諮詢時間更短
- ☐ 患者對自我保健更有信心
- ☐ 患者在不需要時不尋求醫療幫助
- ☐ 患者會盡快接受必要的檢查或治療
- ☐ 減少不必要的檢查
- ☐ 減少不必要的治療
- ☐ 其他:____________________

VII. 總體而言，您會如何描述患者對網路健康資料的體驗？

- ☐ 很好
- ☐ 好
- ☐ 不確定
- ☐ 不好
- ☐ 極差

VIII. 您是否會推薦病患使用本機器學習健康評估網站 MLdoctor

- ☐ 願意
- ☐ 不願意，原因:__________________
- ☐ 沒意見，對醫療資訊與互聯網不熟悉

您的 Email (選填): ______________________________，如有任何相關意見諮詢，可透過 e-mail 聯繫您

謝謝您的寶貴意見，祝您身體健康，事業順利，闔府平安！

ML-Doctor 網站使用滿意度

No: 14

1. 請問您是第一次造訪我們網站嗎？

☑是 ☐否

2. 您的身分是

☐醫師(請完成第 4、5、6 項)
☑臨床醫護人員(請完成第 4、5 項)
☐非臨床醫護人員

3. 年齡:

☐ 18-24
☑ 25-34
☐ 35-44
☐ 45-54
☐ 55-64
☐ 65 以上

4. MLdoctor 網站體驗與觀感調查，請依據問卷題目在方框內打勾

問卷題目	非常不同意	不同意	普通	同意	非常同意
1. 我想我會願意經常使用這個網站。				✓	
2. 我覺得這個網站過於複雜。		✓			
3. 我認為這個網站很容易使用。				✓	
4. 我想我需要有人幫助才能使用這個網站。			✓		
5. 我覺得這個網站的功能整合得很好。				✓	
6. 我覺得這個網站有太多不一致的地方。	✓				
7. 我可以想像大部份的人很快就可以學會使用這個網站。			✓		
8. 我覺得這個網站使用起來很麻煩。	✓				
9. 我很有自信能使用這個網站。			✓		
10. 我需要學會很多額外的資訊，才能使用這個網站。				✓	

5. 醫護人員 MLdoctor 網站體驗調查(如第一項勾選醫師及醫護人員者請繼續作答)

問卷題目	非常不同意	不同意	普通	同意	非常同意
1. 這個網站提供一個合適的管道來傳遞健康照護資訊				✓	
2. 這個網站在我執行健康照護上會有幫助			✓		
3. 這個網站改善了我提供醫療保健服務的途徑				✓	
4. 這個網站讓我更有效率地管理病患的健康				✓	
5. 這個網站讓我跟我的病患溝通更方便			✓		
6. 這個網站讓我有更多機會跟我的病患互動			✓		
7. 這個網站提供一個合適的管道來提供醫療保健服務如: 獲得教育資料，追蹤自己的活動並進行自我評估。					✓

6. 臨床醫師 MLdoctor 網站體驗調查(如第一項勾選醫師請繼續作答)

I. 您每個月的看診人數大約為

☐ 未看診
☐ <20 人
☐ 20-100 人
☐ 100-200 人
☐ >200 人

II. 請預估上個月用網路搜尋健康資訊的病患大約比例為

☐ <1%
☐ 1-2%
☐ 3-5%
☐ 6-10%
☐ >10%
☐ 不確定

III. 您認為網路上的健康資訊的總體品質為

☐ 通常可靠
☐ 有时可靠
☐ 有时不可靠
☐ 通常不可靠
☐ 不知道

IV. 您有任何病患因查閱網路上的資訊而獲得健康上的好處嗎

- ☐ 有
- ☐ 沒有
- ☐ 不確定

V. 您的患者獲得了哪些好處？［可複選］

- ☐ 訂購有用的藥物或其他保健產品
- ☐ 從（聲稱的）實踐者那裡獲得有用的第二意見
- ☐ 獲得有用的風險估計
- ☐ 從病友資訊協助網站獲得有用的建議
- ☐ 儘早尋求適當的醫療幫助
- ☐ 更好地了解他們的狀況
- ☐ 獲得社會對他們狀況的支持
- ☐ 其他:______________________________.

VI. 總體來說，使用網路對您和患者的健康服務有什麼好處？［可複選］

- ☐ 患者更有能力應對症狀或疾病
- ☐ 諮詢時間更短
- ☐ 患者對自我保健更有信心
- ☐ 患者在不需要時不尋求醫療幫助
- ☐ 患者會盡快接受必要的檢查或治療
- ☐ 減少不必要的檢查
- ☐ 減少不必要的治療
- ☐ 其他:__________________

VII. 總體而言，您會如何描述患者對網路健康資料的體驗？

- ☐ 很好
- ☐ 好
- ☐ 不確定
- ☐ 不好
- ☐ 極差

VIII. 您是否會推薦病患使用本機器學習健康評估網站 MLdoctor

- ☐ 願意
- ☐ 不願意，原因:________________
- ☐ 沒意見，對醫療資訊與互聯網不熟悉

您的 Email (選填): ____________________________，如有任何相關意見諮詢，可透過 e-mail 聯繫您

謝謝您的寶貴意見，祝您身體健康，事業順利，闔府平安！

ML-Doctor 網站使用滿意度

No: 27

1. 請問您是第一次造訪我們網站嗎？

☑是　　☐否

2. 您的身分是

☑醫師(請完成第 4、5、6 項)

☐臨床醫護人員(請完成第 4、5 項)

☐非臨床醫護人員

3. 年齡:

☐ 18-24

☐ 25-34

☑ 35-44

☐ 45-54

☐ 55-64

☐ 65 以上

4. MLdoctor 網站體驗與觀感調查，請依據問卷題目在方框內打勾

問卷題目	非常不同意	不同意	普通	同意	非常同意
1. 我想我會願意經常使用這個網站。				✓	
2. 我覺得這個網站過於複雜。		✓			
3. 我認為這個網站很容易使用。				✓	
4. 我想我需要有人幫助才能使用這個網站。		✓			
5. 我覺得這個網站的功能整合得很好。			✓		
6. 我覺得這個網站有太多不一致的地方。		✓			
7. 我可以想像大部份的人很快就可以學會使用這個網站。				✓	
8. 我覺得這個網站使用起來很麻煩。		✓			
9. 我很有自信能使用這個網站。				✓	
10. 我需要學會很多額外的資訊，才能使用這個網站。				✓	

5. 醫護人員 MLdoctor 網站體驗調查(如第一項勾選醫師及醫護人員者請繼續作答)

問卷題目	非常不同意	不同意	普通	同意	非常同意
1. 這個網站提供一個合適的管道來傳遞健康照護資訊				✓	
2. 這個網站在我執行健康照護上會有幫助					✓
3. 這個網站改善了我提供醫療保健服務的途徑				✓	
4. 這個網站讓我更有效率地管理病患的健康				✓	
5. 這個網站讓我跟我的病患溝通更方便				✓	
6. 這個網站讓我有更多機會跟我的病患互動				✓	
7. 這個網站提供一個合適的管道來提供醫療保健服務如: 獲得教育資料，追蹤自己的活動並進行自我評估。				✓	

6. 臨床醫師 MLdoctor 網站體驗調查(如第一項勾選醫師請繼續作答)

I. 您每個月的看診人數大約為

- ☐ 未看診
- ☑ <20 人
- ☐ 20-100 人
- ☐ 100-200 人
- ☐ >200 人

II. 請預估上個月用網路搜尋健康資訊的病患大約比例為

- ☐ <1%
- ☐ 1-2%
- ☐ 3-5%
- ☑ 6-10%
- ☐ >10%
- ☐ 不確定

III. 您認為網路上的健康資訊的總體品質為

- ☐ 通常可靠
- ☐ 有时可靠
- ☐ 有时不可靠
- ☑ 通常不可靠
- ☐ 不知道

IV. 您有任何病患因查閱網路上的資訊而獲得健康上的好處嗎

- ☑ 有
- ☐ 沒有
- ☐ 不確定

V. 您的患者獲得了哪些好處？［可複選］

- ☐ 訂購有用的藥物或其他保健產品
- ☐ 從（聲稱的）實踐者那裡獲得有用的第二意見
- ☐ 獲得有用的風險估計
- ☑ 從病友資訊協助網站獲得有用的建議
- ☑ 儘早尋求適當的醫療幫助
- ☑ 更好地了解他們的狀況
- ☐ 獲得社會對他們狀況的支持
- ☐ 其他:____________________.

VI. 總體來說，使用網路對您和患者的健康服務有什麼好處？［可複選］

- ☐ 患者更有能力應對症狀或疾病
- ☑ 諮詢時間更短
- ☐ 患者對自我保健更有信心
- ☐ 患者在不需要時不尋求醫療幫助
- ☑ 患者會盡快接受必要的檢查或治療
- ☐ 減少不必要的檢查
- ☐ 減少不必要的治療
- ☐ 其他:__________

VII. 總體而言，您會如何描述患者對網路健康資料的體驗？

- ☐ 很好
- ☐ 好
- ☑ 不確定
- ☐ 不好
- ☐ 極差

VIII. 您是否會推薦病患使用本機器學習健康評估網站 MLdoctor

- ☐ 願意
- ☑ 不願意，原因: 在有醫師詢問下使用較好，因為內容不易理解，須輸入之資訊量大.
- ☐ 沒意見，對醫療資訊與互聯網不熟悉

您的 Email (選填): ywerchi@gmail.com **，如有任何相關意見諮詢，可透過 e-mail 聯繫您**

謝謝您的寶貴意見，祝您身體健康，事業順利，闔府平安！

ML-Doctor 網站使用滿意度

No: 30

1. 請問您是第一次造訪我們網站嗎？
☐是　　☑否

2. 您的身分是
☐醫師(請完成第 4、5、6 項)
☐臨床醫護人員(請完成第 4、5 項)
☑非臨床醫護人員

3. 年齡:
☐ 18-24
☑ 25-34
☐ 35-44
☐ 45-54
☐ 55-64
☐ 65 以上

4. MLdoctor 網站體驗與觀感調查，請依據問卷題目在方框內打勾

問卷題目	非常不同意	不同意	普通	同意	非常同意
1. 我想我會願意經常使用這個網站。					✓
2. 我覺得這個網站過於複雜。	✓				
3. 我認為這個網站很容易使用。					✓
4. 我想我需要有人幫助才能使用這個網站。	✓				
5. 我覺得這個網站的功能整合得很好。				✓	
6. 我覺得這個網站有太多不一致的地方。	✓				
7. 我可以想像大部份的人很快就可以學會使用這個網站。		✓			
8. 我覺得這個網站使用起來很麻煩。	✓				
9. 我很有自信能使用這個網站。					✓
10. 我需要學會很多額外的資訊，才能使用這個網站。	✓				

5. 醫護人員 MLdoctor 網站體驗調查(如第一項勾選醫師及醫護人員者請繼續作答)

問卷題目	非常不同意	不同意	普通	同意	非常同意
1. 這個網站提供一個合適的管道來傳遞健康照護資訊					
2. 這個網站在我執行健康照護上會有幫助					
3. 這個網站改善了我提供醫療保健服務的途徑					
4. 這個網站讓我更有效率地管理病患的健康					
5. 這個網站讓我跟我的病患溝通更方便					
6. 這個網站讓我有更多機會跟我的病患互動					
7. 這個網站提供一個合適的管道來提供醫療保健服務如: 獲得教育資料，追蹤自己的活動並進行自我評估。					

6. 臨床醫師 MLdoctor 網站體驗調查(如第一項勾選醫師請繼續作答)

I. 您每個月的看診人數大約為
- ☐ 未看診
- ☐ <20 人
- ☐ 20-100 人
- ☐ 100-200 人
- ☐ >200 人

II. 請預估上個月用網路搜尋健康資訊的病患大約比例為
- ☐ <1%
- ☐ 1-2%
- ☐ 3-5%
- ☐ 6-10%
- ☐ >10%
- ☐ 不確定

III. 您認為網路上的健康資訊的總體品質為
- ☐ 通常可靠
- ☐ 有时可靠
- ☐ 有时不可靠
- ☐ 通常不可靠
- ☐ 不知道

IV. 您有任何病患因查閱網路上的資訊而獲得健康上的好處嗎

- ☐ 有
- ☐ 沒有
- ☐ 不確定

V. 您的患者獲得了哪些好處？［可複選］

- ☐ 訂購有用的藥物或其他保健產品
- ☐ 從（聲稱的）實踐者那裡獲得有用的第二意見
- ☐ 獲得有用的風險估計
- ☐ 從病友資訊協助網站獲得有用的建議
- ☐ 儘早尋求適當的醫療幫助
- ☐ 更好地了解他們的狀況
- ☐ 獲得社會對他們狀況的支持
- ☐ 其他:____.

VI. 總體來說，使用網路對您和患者的健康服務有什麼好處？［可複選］

- ☐ 患者更有能力應對症狀或疾病
- ☐ 諮詢時間更短
- ☐ 患者對自我保健更有信心
- ☐ 患者在不需要時不尋求醫療幫助
- ☐ 患者會盡快接受必要的檢查或治療
- ☐ 減少不必要的檢查
- ☐ 減少不必要的治療
- ☐ 其他:____________________

VII. 總體而言，您會如何描述患者對網路健康資料的體驗？

- ☐ 很好
- ☐ 好
- ☐ 不確定
- ☐ 不好
- ☐ 極差

VIII。 您是否會推薦病患使用本機器學習健康評估網站 MLdoctor

- ☐ 願意
- ☐ 不願意，原因:________________
- ☐ 沒意見，對醫療資訊與互聯網不熟悉

您的 Email (選填): ________________________________，如有任何相關意見諮詢，可透過 e-mail 聯繫您

謝謝您的寶貴意見，祝您身體健康，事業順利，闔府平安！
