# Supplementary material for: Development of an Online Health Care Assessment for Preventive Medicine: A Machine Learning Approach
Source: J Med Internet Res. 2020 Jun 5;22(6):e18585. doi: 10.2196/18585 (PMC7305560; doi:10.2196/18585)
Supplement: Multimedia Appendix 2 [file jmir_v22i6e18585_app2.docx]

Multimedia Appendix Figure 1 The classification and clustering in different stages of chronic kidney disease


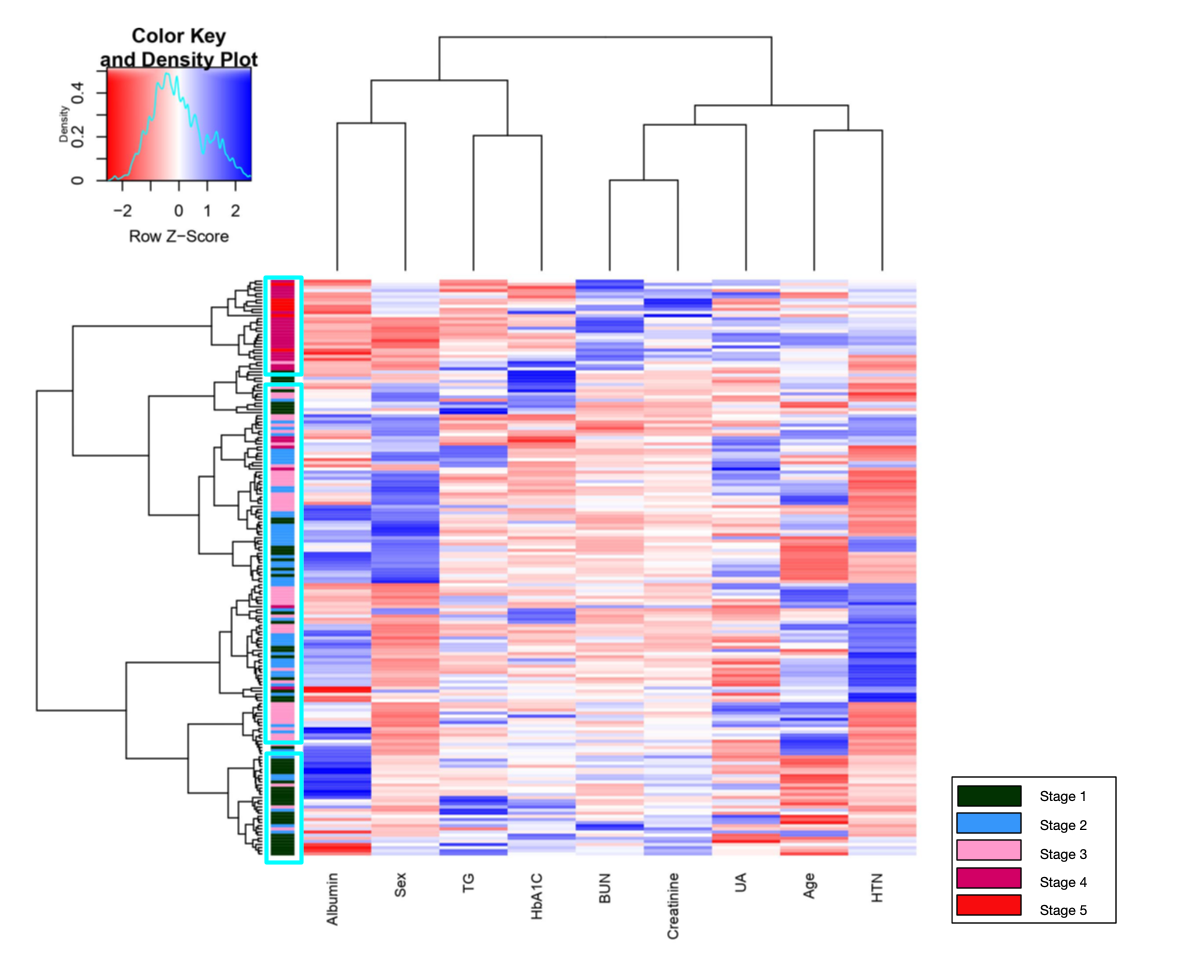


Most of healthy patients in stage 1 (green) is clustered in the bottom of dendrogram. The early CKD patients are grouped in the middle of dendrogram. And the advanced CKD patients are almost clustered in the top of dendrogram. We found that the traditional clinical CKD threshold of eGFR*<*60 ml/min/1.73 m^2^ is not objective since early stage patients groups are staggered arrangement in heatmap. Therefore, preventing the progress of kidney damage is substantial in Preventive Medicine. In fact, we can observe three distinct clusters of CKD patients in our USA validation dataset. Each cluster is marked in light green rectangle. Cluster 1 largely consists of healthy patients; cluster 2 comprises mainly of early stage CKD patients; and cluster 3 mostly includes advanced CKD patients.

Multimedia Appendix Figure 2a The details of stress tests with 100 samples of request


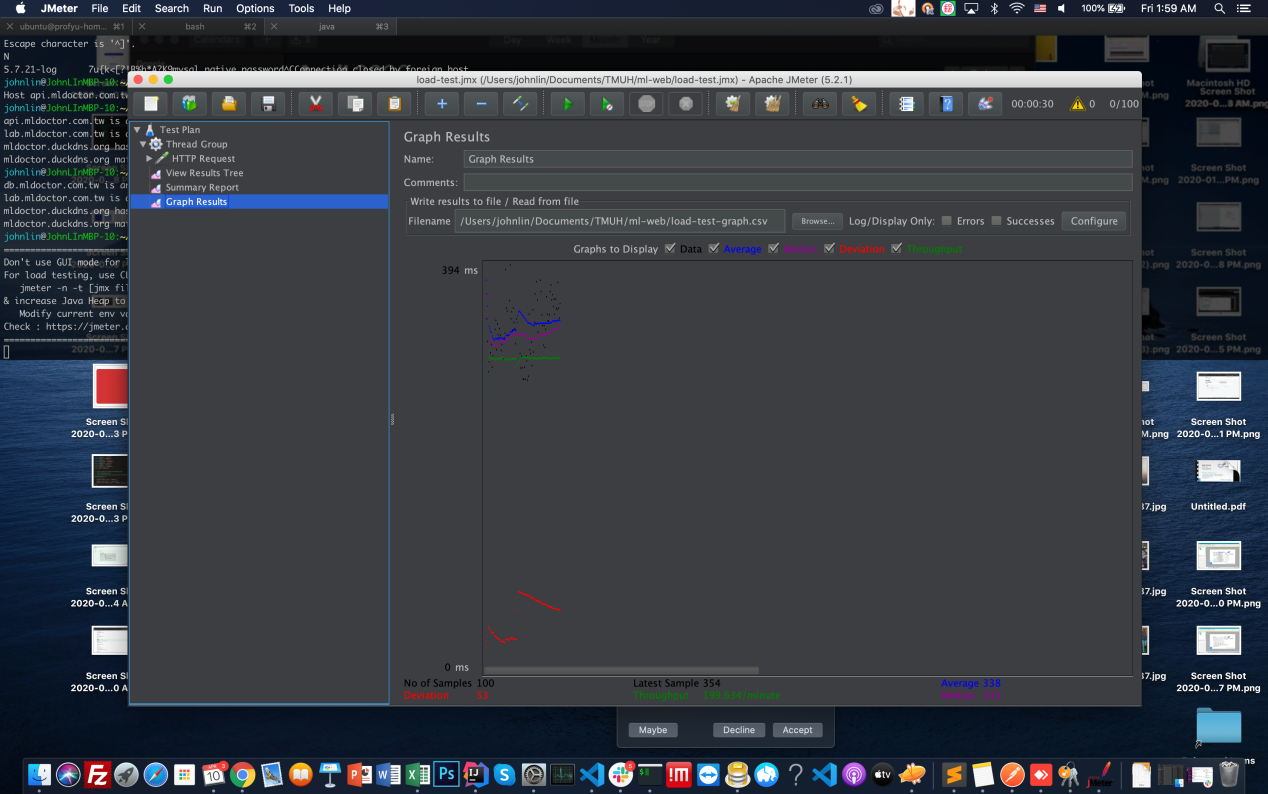


Multimedia Appendix Figure 2b The details of stress tests with 200 samples of request


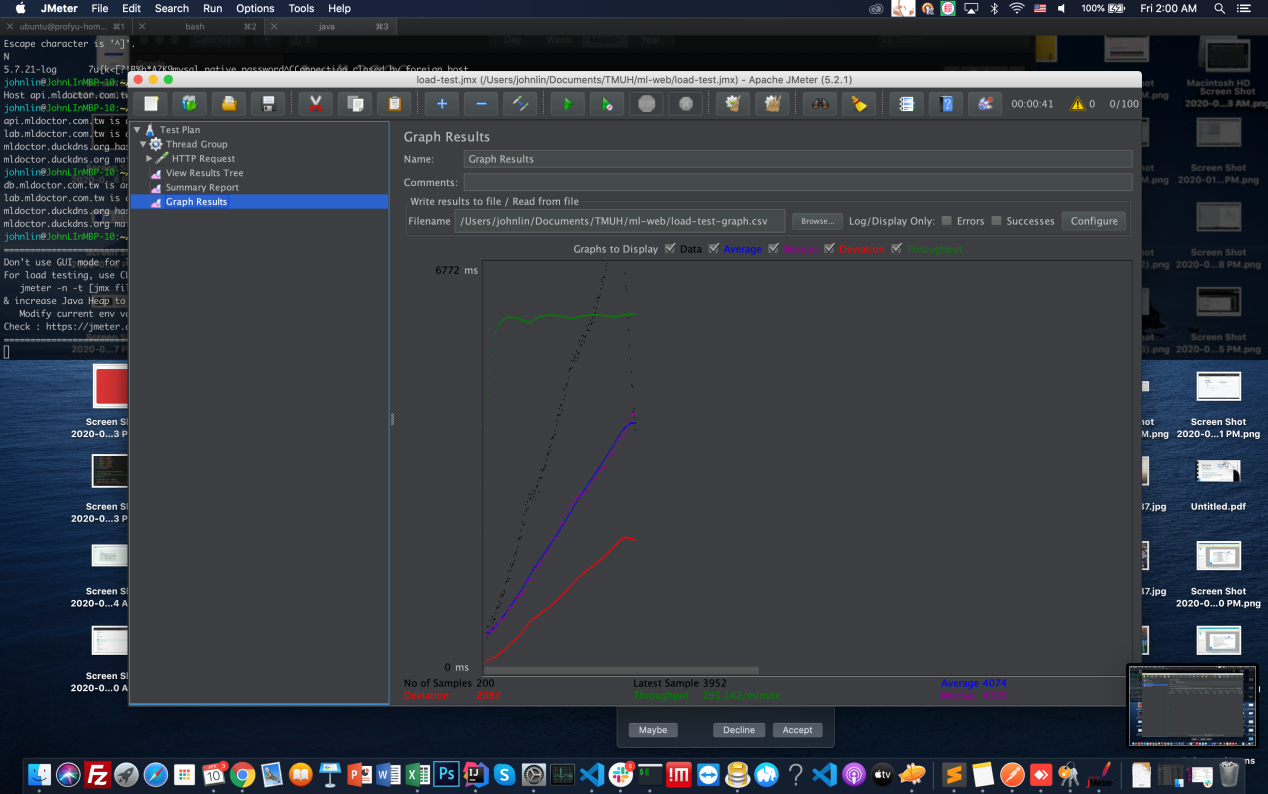


Multimedia Appendix Figure 2c The details of stress tests with 400 samples of request


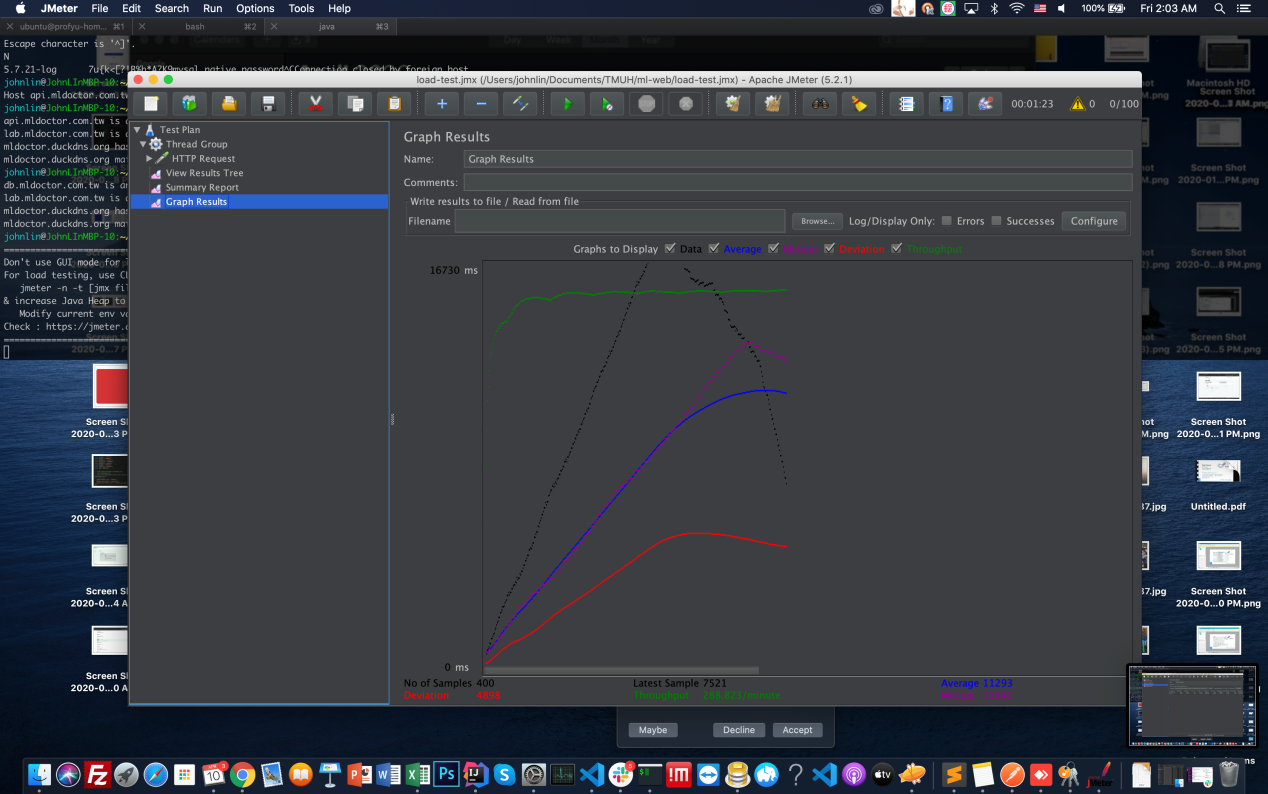


Multimedia Appendix Figure 2d The details of stress tests with 800 samples of request


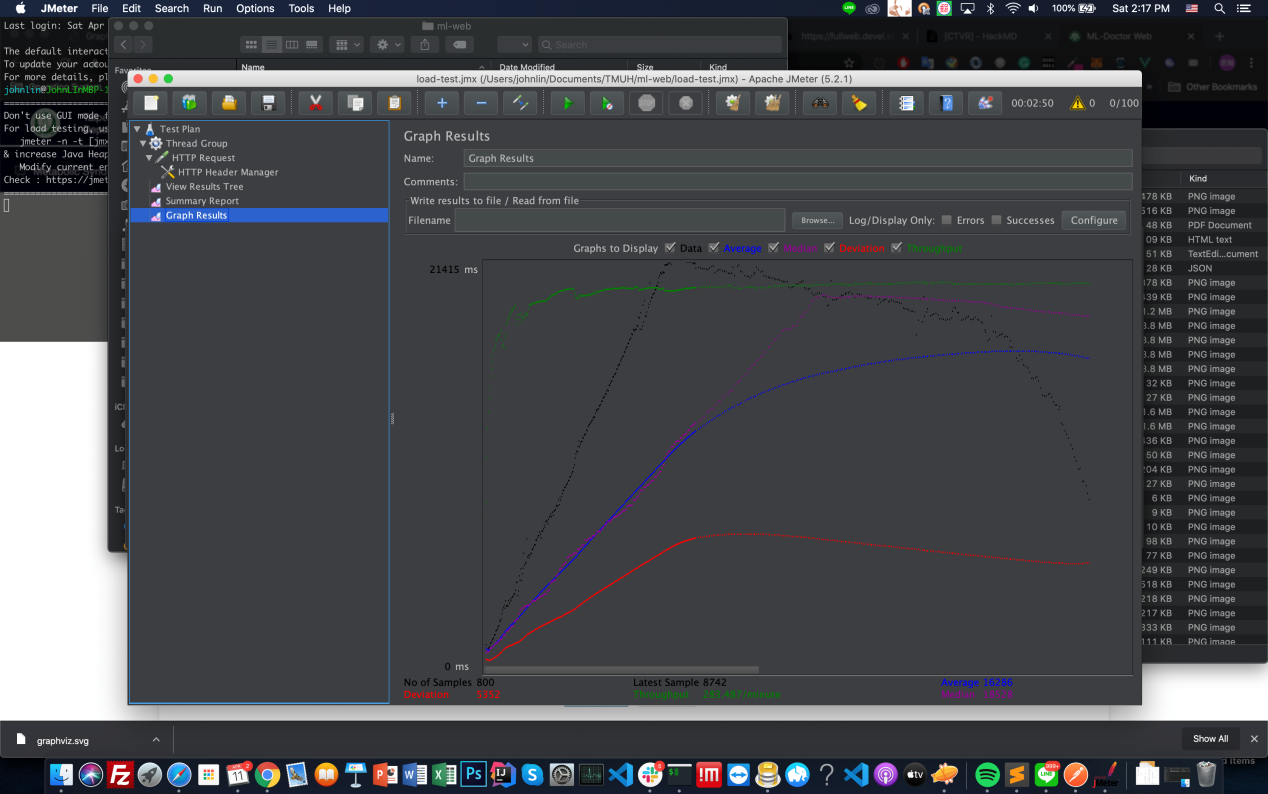


Multimedia Appendix Table 1. The loading test report of system

| Sample size | Average (ms) | Min (ms) | Max (ms) | Std. Dev. | Error (%) | Throughput (per sec) | Received KB/sec | Sent KB/sec |
| --- | --- | --- | --- | --- | --- | --- | --- | --- |
| 100 | 338 | 279 | 653 | 53.42 | 0 | 3.3/sec | 505.32 | 1.44 |
| 200 | 4074 | 435 | 7582 | 2087.97 | 0 | 4.9/sec | 736.95 | 2.10 |
| 400 | 11293 | 432 | 17616 | 4898.65 | 0 | 4.8/sec | 731.08 | 2.08 |
| 800 | 16286 | 544 | 21870 | 5352.92 | 0 | 4.7/sec | 717.58 | 2.04 |
